# Poor Response to Bevacizumab Correlates With Higher IL-6 and IL-8 Aqueous Cytokines in AMD

**DOI:** 10.1167/iovs.65.11.37

**Published:** 2024-09-26

**Authors:** Emma Connolly, Ghaleb El-Farouki, Kiva Brennan, Mark Cahill, Sarah L. Doyle

**Affiliations:** 1 Department of Clinical Medicine, School of Medicine, Trinity College Dublin, Dublin, Ireland; 2 Trinity College Institute of Neuroscience, Trinity College Dublin, Ireland; 3 Research Foundation, Royal Victoria Eye and Ear Hospital, Dublin, Ireland; 4 Progressive Vision Research, Sandyford, Dublin, Ireland

**Keywords:** cytokine, macular edema, age-related macular degeneration, interleukin

## Abstract

**Purpose:**

To evaluate the effect of intravitreal bevacizumab on aqueous levels of a panel of 12 inflammatory cytokines in patients with neovascular age-related macular degeneration (nAMD) and correlate response to treatment, as measured by change in the central subfovea thickness (CST), with cytokine levels.

**Methods:**

Thirty-three treatment-naïve patients with nAMD received a loading dose of intravitreal bevacizumab consisting of three injections at six weekly intervals. The aqueous samples prior to the first (baseline), second (week 6), and third (week 12) injections were analyzed for cytokine levels. Participants were subgrouped based on changes in CST on spectral-domain optical coherence tomography (SD-OCT) at 12 weeks. Group 1 included patients with a decrease in CST (responders; *n* = 27). Group 2 included patients who had no decrease in CST (poor responders; *n* = 6).

**Results:**

Aqueous IL-8 was the only cytokine to demonstrate a significant difference in levels between responders and poor responders, with higher interleukin-8 (IL-8) at week 12 in the poor responder group. Aqueous IL-6 and IL-8 levels showed a positive correlation with CST on SD-OCT (Spearman *r* = 0.45 and 0.55, respectively). There was a temporal increase overall in cytokine concentration accompanying bevacizumab treatment.

**Conclusions:**

Aqueous IL-6 and IL-8 may be important markers of treatment response or poor response in nAMD. Future therapeutic strategies may include targeted treatment against both vascular endothelial cell growth factor (VEGF) and IL-6 and/or IL-8 in patients who do not respond to anti-VEGF treatment alone.

Age-related macular degeneration (AMD) is the most common cause of irreversible vision loss in people over the age of 50 in developed countries, and it accounts for 8.7% of all blindness worldwide. It is estimated that, with the growing aging population in Europe, this number could increase by 13% (77 million people) by the year 2050. The incidence of late AMD is expected to increase to 700,000 cases per year, accounting for 12 million of all AMD cases.^1^ The current standard of care for neovascular age-related macular degeneration (nAMD) is treatment with anti-vascular endothelial cell growth factor (anti-VEGF). Several studies and randomized control trials have established the safety and efficacy of these therapeutics and proven their ability to prevent vascular permeability, thus stabilizing vision loss and improving visual acuity. However, response to treatment is not uniform, with up to 30% of patients failing to achieve these visual gains and a further 2% to 11% of cases displaying tachyphylaxis, or diminished therapeutic response to anti-VEGF treatment following repeated administration over time.^2^^,^^3^ More recently, studies have demonstrated that almost half of anti-VEGF–treated AMD patients have scar tissue after 2 years, with one-third of those presenting with subretinal fibrosis during the first year of treatment.^4^^,^^5^ Development of scar tissue is the most common cause of sustained vision loss. Failure to respond to treatment is known to be influenced by lesion type and disease severity^4^^,^^6^; however, these factors do not fully explain the degree of poor response observed in these studies.

Addressing the limitations of anti-VEGF therapies remains a continued subject of investigation, and the emerging therapeutics strive to improve efficacy and alleviate the treatment burden. These include brolucizumab, a small molecular weight single-chain antibody fragment targeting VEGF-A, and faricimab, a bispecific monoclonal antibody inhibiting both VEGF-A and angiopoietin 2 (Ang-2) that appears to have a synergistic effect in decreasing vascular leakage and inflammation.^7^^,^^8^ However, concerns over the increased risk of occlusive retinal vasculitis and intraocular inflammation associated with brolucizumab have reinforced the need for studies investigating alternative therapeutic targets for treatment of nAMD, including mediators of inflammation and VEGF-independent mechanisms of angiogenesis.

Immune dysregulation of the retina and choroid has been implicated in AMD pathogenesis. Previous studies have investigated the expression patterns of cytokines and chemokines in various biological samples from AMD patients in an effort to understand their association with AMD incidence and progression. Analysis of aqueous humor, which is reported to reflect cytokine levels in the vitreous chamber, has been used to assess the intraocular inflammatory profile in the eyes of patients for several retinal diseases, including AMD.^9^^,^^10^ Furthermore, sampling of aqueous humor from nAMD eyes has suggested that expression of several cytokines such as monocyte chemoattractant protein-1 (MCP-1), interferon gamma-induced protein 10 (IP-10), interleukin-6 (IL-6), and IL-8 are altered following anti-VEGF treatment.^11^ Although initial studies in this area have assessed the cytokine profiles of AMD patients pre- and post-treatment and in comparison to non-disease patients, little is known about cytokine changes over the course of anti-VEGF treatment at the initial loading dose stages or the correlation with anatomical outcomes.^12^^,^^13^

In this study, aqueous humor was collected from a cohort of treatment-naïve patients undergoing six weekly loading doses of anti-VEGF treatment with bevacizumab. The central subfovea thickness (CST) was measured before the loading dose began and at intervals after the loading dose using spectral-domain optical coherence tomography (SD-OCT). Patients were grouped as responders if the CST was reduced, or as poor responders if the CST was unchanged or increased. The rate of change of the 12 cytokines from baseline (BL) to week 12 (W12) and the correlations with CST changes were assessed.

## Materials and Methods

### Study Participants

This prospective study recruited 33 participants diagnosed with nAMD from the Royal Victoria Eye and Ear hospital (RVEEH) in Dublin. Patients received three injections of bevacizumab at 6-week intervals, and all samples were collected over a 6-month study period. All patients underwent full ophthalmic examinations and had full medical histories obtained, including smoking history. This study adhered to the tenets of the Declaration of Helsinki and was approved by St. Vincent's University Hospital ethics committee (WAMD/120815).

### Inclusion/Exclusion Criteria

To be included in the study, patients were required (1) to be over the age of 50 with a new diagnosis of nAMD requiring treatment with intravitreal anti-VEGF; (2) to have had no previous intraocular treatment in the study eye; (3) to have no concurrent ocular co-morbidities; and (4) to be willing to consent to treatment and participation in the study. Exclusion criteria included patients undergoing treatment with corticosteroids or having uncontrolled glaucoma, active inflammation, or diabetic retinopathy.

### Aqueous Sampling

Sample collection and treatment were carried out in a dedicated injection room under aseptic conditions. Three drops of 1% proxymethacaine were instilled over 10 minutes, and one drop of 1% tropicamide was given to dilate the pupil. With the patient lying semi-supine, skin antisepsis was carried out using povidone iodine (5% w/v solution) in the conjunctival fornix and periorbital skin. After a speculum was placed to open the eyelids, aqueous sampling was taken with a 30-gauge needle attached to a 1-mL syringe using a temporal approach medial to the limbus and in a horizontal plane. Approximately 0.05 mL of aqueous humor was obtained and transferred immediately to a –80°C freezer for storage. A 30-gauge needle was used for intravitreal injection of 0.05 mL of bevacizumab either 3.0 mm or 3.5 mm posterior to the limbus.

### Functional Outcome Measurements

At recruitment, all participants underwent a full ophthalmic exam that included visual acuity and OCT. Fundus fluorescein angiography was used to assess choroidal neovascularization activity. OCT was conducted on all eyes at recruitment prior to the first injection and at 12 weeks prior to the third injection. CST was recorded in micrometers using the ZEISS CIRRUS HD-OCT 5000 system (Carl Zeiss Meditec, Dublin, CA, USA).

### LEGENDplex Assay

The concentration of cytokines in the aqueous humor was determined using the LEGENDplex Human Inflammation Panel 1 bead-based immunoassay (BioLegend, San Diego, CA, USA). The assay was prepared as per the manufacturer's instructions, utilizing a BD LSRFortessa Cell Analyzer (BD Biosciences, Franklin Lakes, NJ, USA).

### Statistical Analysis

Data were analyzed using Prism 10 for Windows (GraphPad Software, Boston, MA, USA). Changes in cytokine levels in the full cohort between BL and W12 were assessed using the Wilcoxon matched-pairs signed-rank test. Changes in cytokine levels at BL, week 6 (W6), and W12 in responders or poor responders, as determined by changes in CST, were evaluated using two-way analysis of variance (ANOVA). Samples were analyzed between time points with Sidak's multiple comparison test between responder and poor responder groups and with Tukey's multiple comparison test between time points. Correlation analysis of cytokines with changes in CST (ΔCST) was performed on log-transformed data using Spearman's rank correlation.

## Results

**Figure 1.**
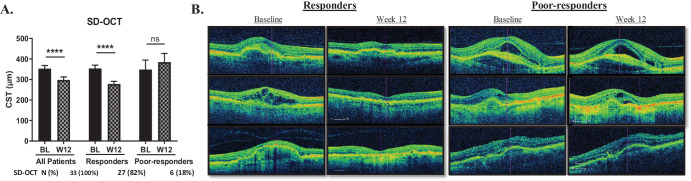
Changes in CST in the clinical neovascular cohort. (**A**) Change in CST from BL to W12 for all participants, including those who responded to treatment and those who had poor response as based on SD-OCT. (**B**) Representative SD-OCT images demonstrating changes in CST from BL to W12 in responders and poor responders. Bar graphs show mean ± SEM analyzed using two-way ANOVA with Sidak's multiple comparison test.

Demographic characteristics of this cohort are summarized in Table 1. Based on changes in CST as measured by SD-OCT, 82% of participants (*n* = 27) responded to treatment with a reduction in CST, whereas 18% of participants (*n* = 6) did not respond to treatment. The average CST for all patients at BL was 352 ± 89 µm, which decreased to an average of 297 ± 86 µm at W12, a change of 55 ± 75 µm (*P* ≤ 0.0001) (Figs. 1A, 1B). There was a significant decrease in CST in participants who responded to treatment, from 353 ± 85 µm at BL to 278 ± 33 µm at W12 following bevacizumab treatment, a reduction of 76 µm (*P* ≤ 0.0001). The participants who were not responsive to treatment had an average CST at BL of 348 ± 113 µm, which increased to 384 ± 105 µm, or 36 µm during the course of treatment (*P* = 0.497).

**Table 1.** Characteristics of Study Cohort (*n* = 33)

Variable	Value
Gender (M/F), *n*	11/22
Age (y), mean ± SD	77 ± 7
Visual acuity, mean ± SD	0.55 ± 0.37
BMI (kg/m^2^), mean ± SD	21.25 ± 1.40
Family history of AMD (yes), *n* (%)	3 (9.1)
Lens status, *n* (%)	
Phakic	24 (72.7)
Pseudophakic	9 (27.3)
Fluorescein angiography, *n* (%)	
Classic	8 (24.2)
Occult	16 (48.5)
Peripapillary	3 (9.1)
Unknown	6 (18.2)
Ophthalmic history, *n* (%)	
Glaucoma	2 (6.1)
Cataract	8 (24.2)
NPDR	1 (3.0)
Smoking status, *n* (%)	
Never	14 (42.4)
Past	10 (30.3)
Current	6 (18.2)
Unknown	3 (9.1)

BMI, body mass index; NPDR, non-proliferative diabetic retinopathy.

Table 2 outlines the mean levels for each cytokine at BL, W6, and W12 in the whole cohort. All cytokines increased following intravitreal injection of bevacizumab, with the exception of IL-12p70, which was virtually unchanged between BL and W12 (*P =* 0.898). A significant increase in levels was observed in cytokines involved in immune cell polarization and chemotaxis, such as interferon‐gamma (IFN‐γ; *P* = 0.0001), IL-23 (*P* = 0.006), IL-33 (*P* = 0.009), IL-17A (*P* = 0.005), and MCP-1 (*P* = 0.009). Drivers of innate immunity and immunomodulation were also found to be increased at W12, including IL-1β (*P* = 0.004), IL-8 (*P* = 0.010), tumor necrosis factor alpha (TNF-α; *P* = 0.010), and IFN-α (*P* = 0.036). Levels of IL-6 and IL-10 were also increased at W12, but not significantly (*P* = 0.073 and *P* = 0.063, respectively).

**Table 2.** Mean Cytokine Concentrations of nAMD Patients at Baseline, Week 6, and Week 12

	BL		W6		W12		
Cytokine	pg/mL	±SEM	pg/mL	±SEM	pg/mL	±SEM	*P* (BL vs. W12)
Innate immune activators							
IL-8	2.54	0.85	2.93	0.79	4.43	0.98	0.010*
IL-6	2.84	0.85	3.22	0.70	6.59	1.77	0.073
IL-1β	9.19	0.21	9.23	0.31	9.97	0.25	0.004**
TNF-α	2.78	0.64	3.70	0.65	4.63	0.63	0.010*
T-cell polarization							
IFN-γ	20.83	4.37	29.66	4.66	42.33	5.10	0.0001***
IL-23	3.57	0.80	5.58	0.96	7.10	1.12	0.006**
IL-33	27.94	9.05	46.52	11.56	54.52	10.91	0.009**
IL-12p70	1.07	0.34	0.85	0.28	0.94	0.33	0.898
Immunomodulation							
IFN-α	2.43	0.42	3.57	0.56	4.52	0.69	0.036*
IL-10	1.88	0.63	2.84	0.72	3.48	0.64	0.063
Chemotaxis							
IL-17A	45.92	9.38	49.98	7.29	69.28	8.19	0.005**
MCP-1	201.95	22.19	221.73	31.33	260.51	23.97	0.009**

* *P* < 0.05, ** *P* < 0.01, *** *P* < 0.001.

**Figure 2.**
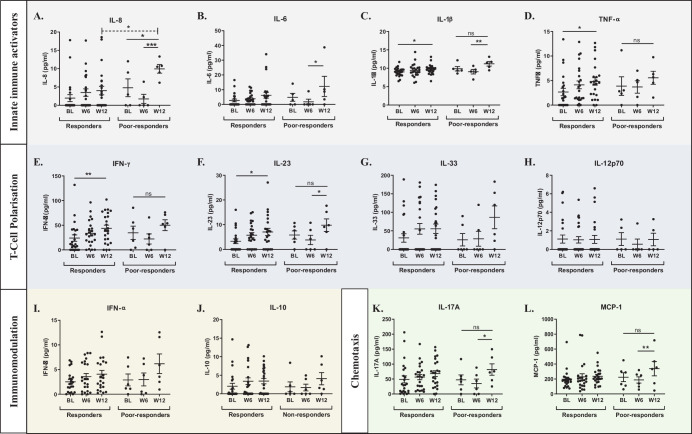
Levels of inflammatory cytokines in patients deemed responders or poor responders as measured by OCT. Patients were grouped by changes in central subfield macular thickness over the 12-week course of treatment measured by OCT. Cytokines were measured in aqueous humor at BL, W6, and W12, and presented grouped by associated function i.e Innate Immune activators, T-Cell Polarisation, Immunomodulation and Chemotaxis. Data are presented as mean ± SEM and were analyzed using two-way ANOVA with Tukey's multiple comparison test for changes between matched samples within each group (*solid line*) and Sidak's multiple comparison test between groups at each time point (*dashed line*).

Figure 2 examines the change in cytokines levels at BL, W6, and W12 in both responders and poor responders to treatment, as determined by change in CST on SD-OCT. Poor responders had significantly higher levels of IL-8 at W12 compared to responders who had a reduction in CST (*p* = 0.045) (Fig. 2A). The poor responder group also had increasing IL-8 between BL and W12 (*p* = 0.041) with a highly significant increase observed between W6 and W12 (*p* = 0.0007), not seen in those who had a reduction in CST.

No other cytokines levels were significantly different between responders and poor responders to bevacizumab therapy. However, there were differences between responders and poor responders in the timing of changes in some cytokine levels during a loading dose of bevacizumab. Responders to treatment demonstrated a steady increase in innate immune cytokines IL-1β (*P* = 0.033) (Fig. 2C) and TNF-α (*P* = 0.032) (Fig. 2D) from BL, with significantly higher levels by W12, which was not seen in poor responders. Increases in these drivers of innate immunity that form the early inflammatory response were delayed in poor responders, with levels only increasing between W6 and W12 for IL-1β (*P* = 0.001) (Fig. 2C) and IL-6 (*P* = 0.027) (Fig. 2B). Similarly, T-cell polarization cytokines IFN-γ (*P* = 0.004) and IL-23 (*P* = 0.013) increased significantly between BL and W12 in treatment responders (Figs. 2E, 2F), whereas poor responders had a significant increase in IL-23 (*P* = 0.047) (Fig. 2F) after W6. Poor responders also showed significantly higher levels of IL-17A (*P* = 0.031) (Fig. 2K) and MCP-1 (*P* = 0.007) (Fig. 2L), cytokines involved in immune cell trafficking and chemotaxis, between W6 and W12, which were not observed in responders to treatment.

**Figure 3.**
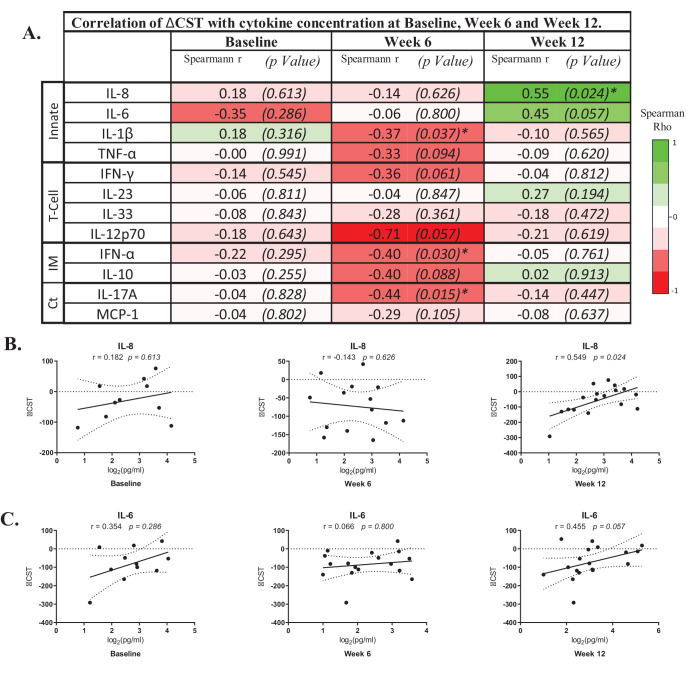
Correlation of ∆CST with cytokine concentration at BL, W6, and W12. (**A**) Summary table of the correlation of ∆CST with cytokine concentrations at BL, W6, and W12. (**B**, **C**) Spearman's rank correlations of log-transformed IL-6 and IL-8 cytokine levels at BL, W6, and W12 with changes in CST between BL and W12. Graphs display regression lines and 95% confidence intervals with Spearman's rho coefficient and associated *P* values. Ct, chemotaxis; IM, immunomodulation; Innate, innate immune activators; T-cell, T-cell polarization.

Correlation analysis of cytokine levels in the cohort as a whole at BL, W6, and W12 and ΔCST from BL to W12 was undertaken (data summarized in Fig. 3A). At BL, there was no significant correlation between ΔCST and any of the 12 cytokines examined. At W6, several medium to strong inverse correlations were seen, particularly for IL-1β (*r* = –0.37, *P* = 0.037), IFN-α (*r* = –0.40, *P* = 0.030), and IL-17A (*r* = –0.44, *P* = 0.015), suggesting that increased levels of these cytokines would be associated with an overall reduction of CST. However, none of these cytokines maintained this correlation by W12. By W12, there was a strong positive correlation between IL-8 (*r* = 0.55, *P* = 0.024) and IL-6 (*r* = 0.45, *P* = 0.057) with increasing CST, indicating that higher levels of IL-8 or IL-6 cytokines correlate with increasing macular edema (Figs. 3B, 3C).

Supplementary Figure S1 outlines the details of the participants’ smoking history. Of the patients who responded to treatment, with a reduction in CST, 52% were non-smokers; 48% of poor responders were current or past smokers. There was no significant differences between any cytokine levels and smoking status.

## Discussion

This study examined the dynamic change in aqueous cytokine response to anti-VEGF suppression in a cohort of treatment-naïve nAMD patients utilizing a multiplex immunoassay. This prospective study was designed to assess primarily how changes in cytokine levels within the first 3 months of bevacizumab treatment correlate with overall changes in macular thickness. We also examined patterns of cytokine responses for indicators of response to treatment.

Our data suggest that, first, in response to anti-VEGF treatment, there is a trend upward in inflammatory cytokine concentration in the aqueous of patients, and, second, the rate of change in cytokine concentration is delayed in patients who do not respond to anti-VEGF therapy (measured by CST), which may potentially be a defining feature of treatment outcome. Fluctuations in aqueous cytokines following anti-VEGF therapy have previously been reported^12^^–^^17^; however, our study is one of the first to indicate a phenotypic response to therapy that may be associated with treatment outcome. Inflammation is a defense mechanism against infection and injury. The presence of proinflammatory cytokines and associated inflammation in eyes with nAMD could be indicative of disease progression, but they may also have a role in the resolution of injury. These data indicate that their continued upregulation following bevacizumab therapy is associated with the observed resolution of macular edema in responders. It is yet to be determined if the delayed response observed in patients with refractory macular edema is due to poor response to bevacizumab therapy or is an inherent limitation in the wound-healing response within these patients.

The most striking association we observed between cytokine levels and failure to respond to anti-VEGF treatment was for IL-8. Out of the 12 cytokines analyzed, only IL-8 levels were significantly different overall between patients who had improved or not improved CST, as higher levels of IL-8 were found in those with increased CST. These clinical data support similar observations in clinical studies of patients with diabetic retinopathy, nAMD, and branch retinal vein occlusion where increased IL-8 is associated with worse macular edema.^16^^,^^18^^–^^20^

IL-8, MCP-1, IL-17, and IL-6 were the only cytokines for which a rise in concentration was observed in the poor responders that was absent in the responders. These four cytokines have all been associated with AMD to some extent in previous reports. IL-8 is known to orchestrate chemotaxis and activation of neutrophils, monocytes, and macrophages, in addition to its role in promoting loss of endothelial barrier integrity and vascular permeability.^21^ It is possible that either or both of these functions could potentially play a role in diminishing responses to anti-VEGF in nAMD.

MCP-1 has been found at increased levels in the aqueous humor of nAMD patients.^22^^,^^23^ It is a cytokine with chemotactic properties similar to those of IL-8, and it has been shown to associate with inflammatory monocyte recruitment and accumulation at atrophic lesions in dry AMD.^24^^,^^25^ Several reports have demonstrated the potential involvement of IL-17 in experimental models of AMD.^26^^–^^28^ IL-17 is known to promote VEGF-mediated angiogenesis by enhancing VEGF-induced growth of vascular endothelial cells,^29^ and it has been shown that IL-17 can stimulate exacerbation of neovascularization in a VEGF-independent manner. Despite finding that higher MCP-1 and IL-17A levels may be observed in the aqueous of patients with nAMD, neither MCP-1 nor IL-17A concentrations correlated with poorer outcome when grouped overall, suggesting that they are not useful indicators of anti-VEGF treatment outcome. We found no significant association between smoking status and cytokine levels at BL or at W12 following anti-VEGF treatment.

Several hypotheses have been suggested to account for poor responsiveness to anti-VEGFs in nAMD. These include loss of drug effectiveness, subtherapeutic dosing or over-extended treatment intervals, and the heterogeneity of nAMD. In this study, patients were presenting for the first time with macular edema and received loading doses of bevacizumab given at 6-week intervals. This is longer than the 4-week interval frequently observed in other studies commencing intravitreal anti-VEGF treatment but was standard practice for newly diagnosed nAMD patients attending the RVEEH in Dublin at the time of sample collection. Therefore, although it is possible that the dose of bevacizumab was suboptimal, it is perhaps more likely that the anti-VEGF resistance we observed is due to the heterogeneity of disease pathology, with bevacizumab-resistant cases reflecting the presence of other pathobiological mechanisms that can influence neovascularization and microvascular exudation that are independent of VEGF.

IL-8 and IL-6 are perhaps the most interesting cytokines in terms of markers for resistance to treatment. Correlation analysis demonstrated that IL-8 was significantly correlated with increasing macular edema, and both IL-8 and IL-6 were the only cytokines to show positive correlations with higher cytokine levels and increased CST (i.e., poorer outcome). These data are in line with previous studies that have implicated both of these cytokines with persistent/recurrent macular edema following intravitreal therapy for nAMD, diabetic macular edema, and increased CST.^30^^–^^33^ IL-6 is not a chemokine, but IL-6 and IL-8 share the ability to promote sustained loss of endothelial barrier function, leading to vascular permeability.^22^ Furthermore, in a previous study, similar in design to ours, Chalam et al.^14^ also reported that higher aqueous IL-6 correlated with poor response in CST in a group of nAMD patients resistant to anti-VEGF treatment. Our data suggest that both aqueous IL-6 and IL-8 may be useful markers of treatment response or resistance in nAMD.

Previous studies have also investigated changes in aqueous cytokines in response to anti-VEGF therapy in treating nAMD,^11^^,^^33^^–^^35^ and diabetic macular edema.^36^ A strength of this study included the inclusion of a treatment-naïve clinical neovascular cohort of patients who attended the clinic at 6-week intervals, and the use of a panel of cytokines. However, several limitations should be noted. First, the human inflammation panel measured by the BioLegend multiplex immunoassay did not include VEGF. Second, although aqueous humor is more accessible and collecting it is safer than collecting vitreous fluid for the assessment of intraocular cytokine profiles, analysis of vitreous fluid may more accurately reflect the cytokine profile within the eye. The low volume of aqueous humor collected during these samplings prevented assessment of technical replicate measurements, and cytokine levels that fall below the lower limit of detection can reduce the number of samples available for analysis.

This analysis identified trends in cytokine levels associated with overall changes in CST from BL and W12. However, this analysis did not take into consideration participants who may have had improved CST if they had been assessed within a shorter time frame. Recent investigations into fluid volumes and dynamics by monitoring OCT changes weekly in the clinic^37^ or through the use of at-home OCT monitoring systems^38^ have noted wide variations in CST reductions over time, particularly with regard to the speed and maintenance of CST reductions, with maximal CST reductions observed at <4 weeks after injection.^37^^,^^38^ It has yet to be determined if cytokine levels measured prior to administering anti-VEGF impact the rate of this short-term response to therapy.

Overall, our data indicate that aqueous IL-6 and aqueous IL-8 may be important markers of treatment response or resistance in wet macular degeneration. Currently, in the United States, the first in-human phase 1 multiple ascending dose study is ongoing using a dual inhibitor antibody biopolymer conjugate (KSI-501) targeting both VEGF and IL-6. Developed by Kodiak Sciences (Palo Alto, CA, USA), the dual mechanism of action of KSI-501 inhibits binding of VEGF-A and placental growth factor (PlGF) to their cognate receptors and soluble IL-6 to both soluble and membrane-bound IL-6 receptors. Although this study is designed to establish the maximum tolerated dose and is enrolling patients with diabetic macular edema, in August 2024 the Phase-III DAYBREAK clinical trial was established to evaluate the efficacy and safety of KSI-501 compared to Aflibercept in patients with nAMD (NCT06556368). Our data support future therapeutic strategies for nAMD that include targeted inhibition of VEGF and IL-6 and/or IL-8 in patients who do not respond to anti-VEGF treatment alone.

## Supplementary Material

Supplement 1
