# Supplementary material for: Poor Response to Bevacizumab Correlates With Higher IL-6 and IL-8 Aqueous Cytokines in AMD
Source: Invest Ophthalmol Vis Sci. 2024 Sep 26;65(11):37. doi: 10.1167/iovs.65.11.37 (PMC11437685; doi:10.1167/iovs.65.11.37)
Supplement: Supplement 1 [file iovs-65-11-37_s001.pdf]

Supplementary Figure 1

A.

Supplementary table 1: Change in CST by smoking status					
Smoking Status	Responders N	(%)	Poor-responders N	(%)	Total
Non-smokers	13	(52%)	1	(20%)	14
Past & Current smokers	12	(48%)	4	(80%)	16
**Total**	**25**	**(100%)**	**5**	**(100%)**	**30**

B.

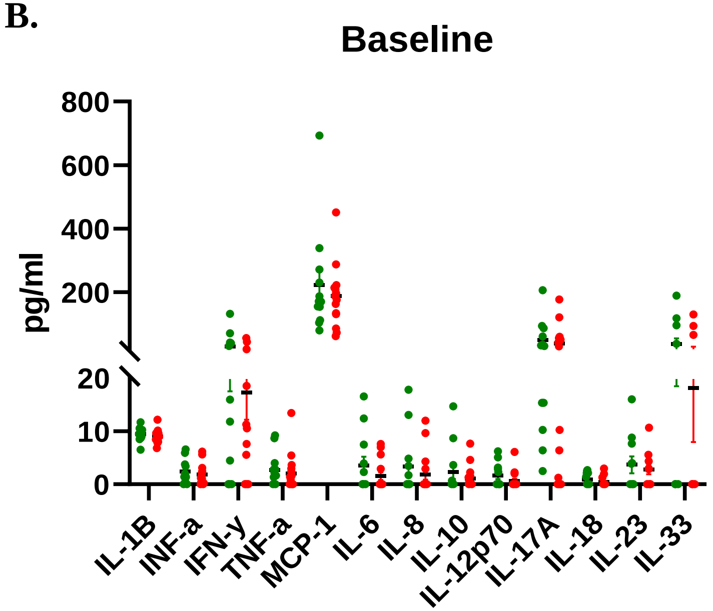

C.

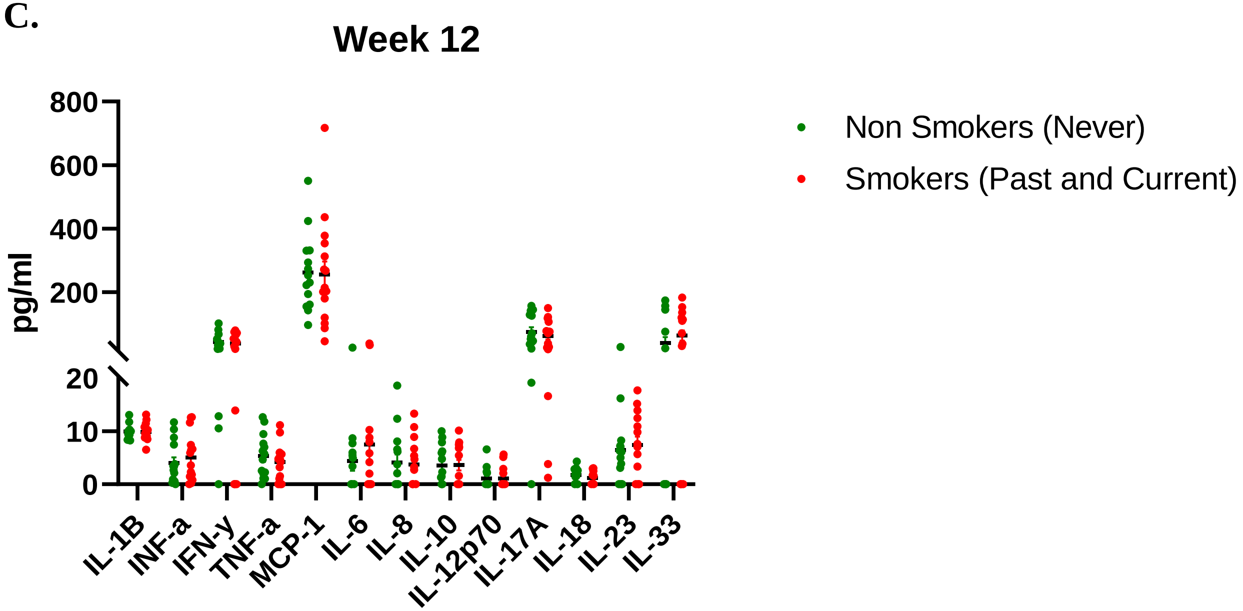

Supplementary Figure 1 Table includes the number of non-smokers and past/current smokers in responders or poor-responders to treatment as based on change in CST measured by OCT **(A).** Mann-Whitney with Holm-Sidak's multiple comparisons test for differences in cytokine levels between non-smokers and current smokers at baseline **(B)** and at week 12 **(C).**
